# Cancer Genomics and Biology 2015 – Meeting Report

**Published:** 2016-01

**Authors:** Louis WC. Chow, Luis Costa, Bin-Tean Teh, Da-Qiang Li, Gu Feng, Xin-Yuan Guan, Asha Nair, Li Zhu, Masahiro Sugimoto, Amit Dutt, Masakazu Toi, Sudeep Gupta, Rajendra Badwe, Stefan Knapp, M. Radhakrishna Pillai, Rakesh Kumar

**Affiliations:** ^1^ Macau University of Science and Technology, Macau, China; ^2^ Jiujiang University, Jiujiang, China; ^3^ Organisation for Oncology and Translational Research, Hong Kong, China; ^4^ Institute of Molecular Medicine, Hospital de Santa Maria – CHLN, Lisbon, Portugal; ^5^ National Cancer Center Singapore, Singapore; ^6^ Fudan University Shanghai Cancer Center and Institute of Biomedical Sciences, Shanghai, China; ^7^ Tianjin Medical University Cancer Institute and Hospital, Tianjin, China; ^8^ University of Hong Kong, Hong Kong, China; ^9^ Rajiv Gandhi Center for Biotechnology, Thiruvananthapuram, India; ^10^ Shanghai Jiaotong University School of Medicine, Shanghai, China; ^11^ Institute for Advanced Biosciences at Keio University, Keio, Japan; ^12^ Tata Memorial Centre, Advanced Centre for Treatment, Research and Education in Cancer, Mumbai, India; ^13^ Kyoto University Graduate School of Medicine, Kyoto, Japan; ^14^ Organisation for Oncology and Translational Research, Kyoto, Japan; ^15^ Structural Genomic Consortium, University of Oxford, Oxford, UK; ^16^ Johann Wolfgang Goethe-University, Frankfurt, Germany; ^17^ School of Medicine and Health Sciences, George Washington University, Washington, DC, USA

**Keywords:** genome biology, transcriptome, epigenetics, cancer stem cells, cancer therapeutics

## Abstract

The Cancer Genomics and Biology 2015 meeting embodied a three way collaboration among colleagues from the Global Cancer Genomics Consortium (GCGC), the Unifaith Cancer Institute China and Jiujiang University of China. The meeting marks the fifth and the last meeting of GCGC, which was formed in 2010. Previous four GCGC meetings have been held at the Tata Memorial Center- Mumbai, Institute of Molecular Medicine-Lisbon, and Graduate Medical School Kyoto University-Kyoto. In contrast to the genomic themes of the previous meetings, the 2015 conference theme was at the interface of laboratory and translation research and emerging therapeutics as reflected in the shared interests of all three collaborative entities – Cancer Genomics and Biology 2015. This year's conference was co-organized by the Jiujiang University at the Run Run Shaw building, Jiujiang University, Jiujiang City, China, on November 13 and 14, 2015. The conference attracted over 174 participants with 13 platform presentations. Scientific sessions included a plenary and five platform scientific sessions with themes ranging from biomarkers, stem cells and markers of the tumor microenvironment, proteomics and epigenetics, big data, to hormone and expression profiles. The meeting concluded with closing remarks by conference co-chairs emphasizing with the need to broaden membership across the globe, establishing priorities, and redrafting a white paper to launch a new consortium.

## INTRODUCTION

The recently concluded Cancer Genomics and Biology 2015 meeting (CGB 2015) embodied a meaningful collaboration among colleagues from the Global Cancer Genomics Consortium (GCGC), the Unifaith Cancer Institute and Jiujiang University from China. The meeting was particularly rewarding as it was to mark the 5^th^ consecutive annual GCGC meeting but this time by spreading its wings to China by promoting collaborations in Cancer Genomics and Biology through three-ways partnership with the Unifaith Cancer Institute and Jiujiang University. The GCGC is an international organization founded by scientists, cryptographer, physician scientists and oncologists from five institutions – the School of Medicine and Health Sciences of the George Washington University, USA; the Kyoto University Graduate School of Medicine, Japan; the Rajiv Gandhi Center for Biotechnology, India; the Tata Memorial Centre, India; the Institute of Molecular Medicine, Hospital de Santa Maria, Portugal; and the Structural Genomic Consortium at the University of Oxford, UK.

Previous four GCGC meetings have been held at the Tata Memorial Center- Mumbai, Institute of Molecular Medicine-Lisbon, and Graduate Medical School Kyoto University-Kyoto. The CGB 2015 was also special as it represented the last meeting of the GCGC which was formed in December 2010 for a period of five years. The meeting was also part of a new chapter with additional colleagues from around the globe by relaunching a modified consortium in 2016 to reflect changing horizons, the nature of scientific questions and available shared resources, and professional interests of our colleagues. This year's conference was co-organized by the Jiujiang University at the Run Run Shaw building, Jiujiang University, Jiujiang City, China, on November 13 and 14, 2015. The conference attracted over 10 overseas and 164 Chinese domestic participants and included 13 invited platform presentations.

### Focus on the Cancer Genomics, Biology and Cancer Patient Care

In contrast to the genomic themes of the previous meetings, the 2015 conference theme was at the interface of laboratory and translation research and emerging therapeutics as reflected in the shared interests of all three collaborative entities – Cancer Genomics and Biology 2015. The conference was opened by its Co- Chairman Professor Yao-fang Yang, Vice President Jiujiang University with a brief synopsis of the academic and scholarly activities and clinical medicine at the University. Prof. Yang also introduced the other two conference Co-Chairs Professor Louis Chow and Professor Rakesh Kumar, and highlighted the role played by newly developed research methodologies and cancer biology insights in medical education, training, research and clinical care at the Affiliated Hospital of Jiujiang University. Scientific sessions included a plenary and five platform scientific sessions with themes that included: 1) biomarkers; 2) two sessions on stem cells and markers of the tumor microenvironment; 3) proteomics and epigenetics; 4) big data; and 5) hormone and expression profiles. The meeting was concluded with closing remarks by Professors Chow, Kumar and Yang, and a discussion about the changing mission of the consortium, the need to integrate additional oncologists and cancer scientists across the world by re-drafting a consortium white paper, and discussed the venue and host of the 2016 meeting.

### Biomarkers

The meeting started with an opening plenary presentation on “Prognostic biomarkers in metastatic breast cancer” by Professor Luis Costa from Lisbon Medical Academic Center Portugal. Dr. Costa started by highlighting the value of understanding the most important limitations in the treatment of breast cancer - the development of resistance against most commonly used endocrine and HER2-directed therapies and associated predictive biomarkers. He presented data from the BOLERO-2 study showing that the combined use of mTOR inhibitor everolimus and exemestane in second line treatment of aromatase inhibitor resistant breast cancer lead to an increased time to progression, suggesting reversal of resistance. He also shared his own data showing a positive correlation between the levels of pS6 kinase, a biomarker of mTOR activity, with survival in metastatic BC patients [1]. Per Dr. Costa, inhibition of CDK4/6 by palbociclib is also effective in reversing or preventing endocrine resistance and increasing time to progression in metastatic breast cancer patients upon combining with the aromatase inhibitor letrozole. However, he pointed out that we do not have yet a biomarker to select the patients for CDK4/6 inhibition. Regarding resistance to HER2- directed trastuzumab therapy, Dr. Costa reminded the audience of the unprecedented results of the CLEOPATRA study (i.e. superior overall survival when pertuzumab is added to trastuzumab and docetaxel) and the efficacy of T-DM1 for trastuzumab and lapainib resistant disease. Dr. Costa was also excited about the recently reported association of the high levels of stromal tumor-infiltrating lymphocytes with the lack of benefit from adjuvant trastuzumab but wondered about the validity of this finding in metastatic patients. Besides he shared recent findings from his lab showing an association between RANK's missense SNPs and prognosis of breast cancer patients with bone metastases and suggested an increased overall survival of patients with homozygous polymorphisms compared to those with heterozygous ones [2]. Dr. Costa closed his presentation by emphasizing that there is a need to support clinical decisions with biomarkers as predictive indicators of treatment response or resistance.

Professor Rakesh Kumar from the George Washington University started his plenary lecture by elaborating on the significance of extra- and intra-cellular milieu in modifying the genome through chromatin remodeling and specifically discussed the value of targeting the MTA1 master chromatin remodeler, one of the most frequently up-regulated oncogenes in human cancer [3]. MTA1 up-regulation correlates with an aggressive and invasive tumor phenotypes and unfavorable outcome for cancer patients. At the cellular level, MTA1- containing chromatin remodeling complexes regulate a range of processes including, cell survival, invasiveness, transformation, DNA-damage response, angiogenesis, metastasis and therapeutic sensitivity of tumor cells. Dr. Kumar also discussed the role of MTA1-containing chromatin remodeling complexes as a hub to modify the expression of genes with functions in embryonic stem- cell differentiation, reprogramming of pluripotent stem cells and mesenchymal as well as cancer stem cells. Furthermore, Dr. Kumar shared recent literature about the contribution of MTA1 in modifying the chemo-sensitivity of cancer cells in experimental model systems. Dr. Kumar also presented a brief summary of advances in the past two decades since the discovery of MTA1 and shared his view about the yet to be realized progress in the area of MTA1 in cancer biology and treatment. He concluded his presentation by highlighting the roles of signal- dependent combinatorial post-translational modifications of MTA1 in determining the nature of resulting MTA1- contaning chromatin remodeling complexes and selection of downstream target genes [4]. Finally, he discussed how selective recognition or targeting of such post-translational modifications of MTA1 could serve as superior biomarkers and targets in cancer.

Professor Da-Qiang Li from the Fudan University Shanghai Cancer Center highlighted the role of the ring- between-ring (RBR) family of E3 ubiquitin ligases in breast cancer progression. He found that one of ring- finger proteins (RNFs) is significantly down-regulated in human breast tumors as compared to its level in the matched adjacent noncancerous tissues, and predicts a better survival of breast cancer patients. Further analysis revealed that this molecule is largely silenced by DNA methylation of the putative CpG island and that treatment with the DNA methylation inhibitor or knockdown of the methyl-binding protein 4 (MBD4) activates the expression of this RNF. The remainder of Dr. Li's presentation was focused on the underlying the nature of its downstream substrate(s) that might be responsible for the noted mechanism of action of this RNF in human cancer. Interestingly, Dr. Li was able to examine the status of this RNF as well as its putative substrate in primary breast cancer patients. Dr. Li concluded his presentation by establishing the significance of epigenetically silencing of a ligase in breast cancer progression and the value of various components of this pathway as biomarkers.

### Stem Cells and Markers of Tumor Microenvironment

The highlights of the session included insightful discussion on the latest advances in Asian Cancer Genomics and their implication in our understanding and management of human cancer by Dr. Bin-Tean Teh from the National Cancer Center Singapore. The core of Dr. Teh approach resides in high-throughput technologies to ask complex yet relevant cancer genomic questions using the appropriate tumor specimens, and take these findings back to the patients by correlating them with clinicopathological variables and discover new therapeutic targets and biomarkers for the benefits of cancer patients. Dr. Teh used fibroepithelial breast tumors as an example to illustrate the approach taken by his team and how they discovered MED12 and RARA as frequently mutated genes in this group of tumors [5]. The second part of Dr. Teh's presentation was focused on the genetics of pathogen- or no-pathogen driven cholangiocarcinomas, and its impact on pathogenesis of the disease [6]. In the last segment of his presentation, Dr. Teh shared their very exciting studies on herbal carcinogenesis based on the association of Aristolochic Acid and the development of upper urinary tract urothelial cancer in certain Asian population. Finally, Dr. Teh highlighted the need to start using genomic biomarkers for screening individuals who might be susceptible to certain cancers.

Dr. Feng Gu from the Tianjin Medical University Cancer Institute and Hospital discussed the role of tumor microenvironment in tumor initiation and progression via supporting the cell proliferation, self-renewal capacity, immune evasion, and apoptotic resistance. In addition, he pointed out that the research of tumor should focus on a combined mechanism including the tumor microenvironment. Dr. Gu also discussed the merit of using a 3-D tumor model over 2-D cell culture system to simulate the tumor microenvironment using primary culture systems. He finished the discussion by focussing on multifactorial contribution of the network of tumor cell signaling and extracellular signaling in regulating cancer cell migration and metastasis.

Dr. Asha Nair from the Rajiv Gandhi Centre for Biotechnology Trivandrum discussed the contribution of cancer stem cells in the recurrence of colorectal cancer patients who have undergone surgical resection. The focus of her presentation was on the surgical margins and how one best can decide the optimal distal margin to be removed to minimize tumor recurrence. She argued that “a negative distal margin could be crucial for reducing risk of local and distant recurrence and post-surgery life of CRC patients.” In this context, Dr. Nair explored the molecular signatures and biomarkers that could be potentially used to make a decision about the resection length. Data presented suggest the feasibility of collecting enriching cancer stem cell population, i.e. CD133, CD44, EpCAM positive, from clinical CRC specimens through FACS. The team found that distal surgical margins are enriched with EpCAM/CD133 positive cells as compared to normal counterpart. Interestingly such cells in distal surgical margins also express drug resistance genes in some cases. Dr. Nair closed her presentation by suggesting that distal tumor margins may represent a safe niche for cancer stem cells and drug resistance cells – both important in disease recurrence. Therefore, Dr. Nair shared her experience in providing critical biomarker-based information to surgical oncologists to make an informed decision of the surgical length to be removed as they operate on CRC cancer patients.

Professor Xin-Yuan Guan from The University of Hong Kong presented a fascinating example of reversal of non-cancer stem cells into cancer stem cells by reprogramming through ATOH8 atonal helix-loop-helix transcription factor. He noted an overall ATOH8 down- regulation in hepatocellular carcinomas and these events are associated with poor differentiation and disease- free survival, and carried out functional study to show a tumor suppressive nature of ATOH8 [7]. Dr. Guan found that ATOH8 may be a board repressor of stemness- associated genes such as OCT4, NANOG and CD133, and accordingly, the loss of ATOH8 in CD133+ cancer stem cell population with ability to self-renew, differentiate and resist chemotherapy. Consistent of these observations, Dr. Guan presented additional evidence showing that ATOH8 depletion may lead to a better generation of induced pluripotent stem cells. In brief, Dr. Guan suggested that CD133 negative cells could be reprogrammed to destine into CD133+ cancer stem cells by modifying the levels of ATOH8; and conversely, ATOH8 expression may enhance the sensitivity of liver cancer cells to chemotherapy.

### Proteomics and Epigenetics

Professor M. Radhakrishna Pillai from the Rajiv Gandhi Centre for Biotechnology highlighted the role of chemotherapy-induced emergence of drug resistance cells with cancer stem cells properties in controlling the therapeutic response versus failure. Dr. Pillai's approach was focused on studying the biology of persister cells - surviving cell population after extremely high doses of chemotherapy. It appears that persister cells exhibit an increased autophagy and mitophagy as well as constitutive activation of redox masters. Dr. Pillai shared data establishing a quick shift in low proteasome activity at the onset of autophagy and that chronic mitophagy facilitate long term survival of resistant cells in low nutrient condition and intracellular protein stress. Over time, this population led to the generation cell populations with heterogeneous ROS and distinct tumorigenic potential. The second part of Dr. Pillai's presentation was focused on understanding the dynamics of genes and proteins during the evolution of drug-resistance through decisive stages. Results from proteomics and transcriptomics analyses suggested that each cell state transition is generally marked with an unique cascades of signaling molecules and unusual stem cell markers, such as CD36, CD68, CD70 and heat shock proteins. Dr. Pillai suggested that increased intracellular inflammatory signaling simultaneously triggers autophagy dependent damage clearance during long cell cycle arrest while heat shock proteins and ubiquitin dependent pathway subsequently ensures emergence of few cells with altered intracellular protein network in delayed manner.

Professor Li Zhu from the Shanghai Jiaotong University School of Medicine shared her experience in stratifying breast cancer patients based on the “primary prognostic predictors” such as tumor size, grade and lymph node status, HER2, estrogen receptor, and progesterone receptor (PgR) at the time of diagnosis. Dr. Zhu also discussed the evolution of the “secondary prognostic assays” which are expected to provide a better predictions of clinical outcome than the traditional clinical and pathological standards, because around 50% of all recurrences in ER+ cancers develop after 5 years, Dr. Zhu discussed the prognostic value of the newer tests (Prosigna™, EndoPredict™ and BCI™) for late recurrences and identify specific group of patients might benefit from extended endocrine therapy. Dr. Zhu also shared the excitement associated with a recent gene expression-based outcome predictor for adjuvant chemotherapy and endocrine therapy sensitivity as the first example of a tertiary residual risk predictor. In brief, Dr. Zhu highlighted the significance of step-wise utilization of primary, secondary and tertiary prognostic endpoints in the treatment of breast cancer.

### Big Data

Professor Stefan Knapp from the University of Oxford and Johann Wolfgang Goethe-University discussed recent developments targeting bromodomains. In the first section of the talk he provided an overview on BET bromodomain inhibitors and the potential of these protein interaction inhibitors in the oncology field [8, 9]. After this introduction he selected examples of non- BET bromodomain inhibitors in particular inhibitors that target bromodomains present in SMARCA2/4 as well as CBP/EP300. Both of these bromodomain inhibitors are non-toxic to cancer cells. However, inhibition of SMARCA2/4 bromodomains mediates expression of key genes important for certain cell differentiation. Interestingly, CBP/EP300 bromodomain inhibition affects leukemia inducing cells (LICs) resulting in differentiation and induction of apoptosis. In addition, CBP/EP300 bromodomain inhibitors are synergistic with doxorubicine as well as BET inhibition suggesting that combination studies or CBP/BET dual inhibitors may lead to better efficacy in leukemia and potentially in other cancers.

Dr. Masahiro Sugimoto from the Institute for Advanced Biosciences at Keio University presented his work about the development of a mathematical algorithm which could be used to predict the outcome of neoadjuvant chemotherapy for HER2 positive breast cancer and how he got involved in such research questions. Neoadjuvant chemotherapy is generally used the reduction of tumor size, and pathological complete response (pCR) post- neoadjuvant chemotherapy is viewed as a prognosis marker. Dr. Sugimoto highlighted the poor predictive accuracy of pCR in neoadjuvant setting using trastuzumab, and the need to develop a novel mathematical model based on artificial intelligence to realize better accuracy than conventional statistical approaches. He also discussed the steps taken to validate the model using a dataset involving 776 breast cancer patients. He shared his hope that such a model would contribute to the personalization of the neoadjuvant chemotherapy treatments.

### Hormone Expression Profiles

Professor Louis Chow from the Macau University of Science, Technology and Organisation for Oncology and Translational Research and Jiujiang University discussed emerging targeted treatment modalities for breast cancer [10]. He started by comparing the traditional, clinical risk factors for breast cancer patients with several novel molecular arrays that may help determine recurrence risk. Dr. Chow provided an overview on the current different risk-arrays, largely for luminal breast tumors. PAM-50™, Oncotype DX™, Mammaprint™, BreastPRS™, EndoPredict™ and Mammostrat™ are all breast cancer risk assessment arrays and Dr. Chow inter-compared these different arrays for gene panels, methodology, timing and patient selection to optimise array utilization. Dr. Chow continued to discuss breast cancer subtypes, noting that Triple Negative Breast Cancers (TNBC) have recently been further sub classified into six distinctly separate subtypes, responsive to different classes of drugs. TNBC should no longer be regarded as one molecular group. Dr. Chow continued his presentation by highlighting the need of better methods to monitor tumor molecular changes over time and discussed the liquid biopsy, thereby stressing tumor heterogeneity. He ended his presentation by seeking a better bi-directional interactions between clinicians and laboratory scientists and continuing the development of technologies such as liquid biopsies.

Dr. Amit Dutt from the Tata Memorial Centre discussed the nature of genomic and molecular changes associated with the events in the surrounding tissues of breast cancer patients undergoing surgery. The study was undertaken to address a broader question about the transcriptomic status of breast tumors in patients who opt to undergo surgical intervention which is also known to trigger perioperative hypoxia. Dr. Dutt started with the study design involving transcriptomic analyses of breast tumors collected at distinct presumed hypoxic stages. He also discussed the status of various regulatory networks as emerged from the study. Dr. Dutt finished his lecture by suggesting that targeting one or more of these nodules during perioperative surgery may influence the clinical outcome of breast cancer patients undergoing surgical intervention.

## CONCLUSIONS

In summary, the conference was two full days of scientific celebration of ideas and discussions of recent advances in cancer genomic biology, therapeutics, and treatment. Most of the questions and answers segments were dominated by discussing how to take forward the findings to the bedside and what should be done to maintain the momentum gained at this and preceding four meetings. Another distinctive feature of the meeting was an active participation of medical educators and professors and medical students. It was not uncommon to witness questions from practicing clinicians and educators relating the scientific presentations with their teaching and training responsibilities. Keeping in-line of the past four meetings, issues surrounding the value of tumor heterogeneity and transcriptome and how to design start anti-cancer agents echoed throughout the conference. As expected, scientific sessions started with an overview of the field, what's emerging, what are the current limitations, and how one can take the research question forward in the overall interest of cancer biology and treatment. Finally, in the closing remarks by conference Co-Chairs, the group discussed about the nature of feasible activities within the changing framework of consortium members and use of tele-medicine for the benefits of cancer patients.
